# Supplementary material for: Determinants of practice location choices among physicians and medical students in Mali: Insights into addressing medical deserts through evidence-based strategies
Source: PLOS Glob Public Health. 2025 Jun 25;5(6):e0004399. doi: 10.1371/journal.pgph.0004399 (PMC12193920; doi:10.1371/journal.pgph.0004399)
Supplement: Attachment — Submitted filename: Ansiima Sheillah Peer review.docx [file pgph.0004399.s001.docx]

**Reviewer’s report**

**Study title:** Determinants of practice location choices among physicians and medical students in Mali: insights into addressing medical deserts through evidence-based strategies

**Version:** 1.0	**Date:** 24^th^ March 2025

**Reviewer:** Sheillah Ansiima

**Summary of research and overall impression**

This paper describes the determinants of practice location choices among physicians and medical students in Mali. It should be publishable provided some extra work is done on the results and discussion section. The methods section and presentation of the results were not satisfactorily done and the interpretation of the results did not provide adequate depth. Additionally, there was no consistency in terminology with different terms being used interchangeably to refer to the same concept. There is a disparity where the authors use the term physician in the title and the term doctors throughout the study. The authors should rewrite their article to use either of the mentioned terms ie either physicians or doctors.

**Major Revisions**

**Abstract:** There is a repetition of the study aim in the abstract. In sentence 3 it is mentioned that “this topic remains underexplored, motivating us to conduct this study to identify factors influencing doctors' workplace decisions in Mali.” While sentence 9 states that “This study elucidates doctors' motivations and identifies key factors associated with their choice of practice location.” The data analysis methods used are not mentioned in the abstract. Additionally, the main findings of the study were not well articulated.

**Introduction**

Overall, the introduction does not properly highlight the problem that led to the conduct of the study. The authors should rewrite the article to articulate the problems that arise as a result of a medical desert.

The definition of the term medical desert is not referenced. The authors should rewrite this to reference related literature.

In paragraph 2, the authors state that “Nationally, only 32% of these centers have a physician, compared to 98% in Bamako, while the figure drops to just 29% in the rest of the country” Does this mean that only 32% of community health centers have a physician. And of these physicians 98% work in Bamako? What does the 29% in the rest of the country mean? These statistics are not clear and make it harder for the reader to understand the message.

**Methods**

**Study design:** The authors mention that the study is a cross-sectional study in Mali however it is unclear whether the study was a nationwide study or a study among specific community health centers.

**Sampling:** There were two distinct categories of participants, was simple random sampling the most appropriate sampling method for this study?

**Data collection:** The authors do not mention the type of questionnaires used. Furthermore, the authors do not mention whether the pre-tested students and physicians included in the pre-test were included in the final study.

**Statistical analysis:** The authors do not mention whether the data was cleaned. The model of R software used is also not mentioned. Furthermore, the authors do not describe how the variables were selected for multivariate analysis. It is important that the authors rewrite this section to make it more precise.

**Results**

Overall, the narratives presented do not adequately reflect the tables presented. There was also some lack of consistency in the description of the results for example some column headings did not reflect what the column values presented. Additionally, some numerical values were presented as whole numbers while others included decimal numbers.

In the methods sections the dependent variable is named “choice of practice location”, the table uses “practice location” while the narrative is “preferred practice location”. Do these mean the same thing?

**Results Paragraph 1:** The authors mention the following variables however these are not presented in the table namely; age and professional experience. The authors further mention that “among the 358 respondents, 191 (53.35%) were born and/or raised in urban areas” yet the table has a variable named “rural origins”, do these mean the same thing?

**Results Paragraph 2:**

The authors state that “When asked about their preferred practice location, less than 38% (135/358) of respondents indicated a preference for working in rural areas if given the choice”. Yet the results in the table for practice location reflect 22% as the value for preference for working in rural areas. Why did the authors choose to use 38%?

Table 1 reflects 68% as the percentage representing physicians yet the authors state that “physicians constituted over 68%”. Additionally, the “77% of the physicians working in the private sector” is not reflected in table 1.

The percentages presented in the statement “Bamako was the practice location for 51.22% of respondents, while the remaining 48.78% worked in other regions” do not represent the findings presented in the table.

For the statement “More than 69% of respondents reported no prior experience working or interning in rural settings” why didn’t the authors use the actual percentage presented in the table?

**Table 1**

The percentages presented for all variables do not add up to 100%. The authors should revise the table.

**Table 2**

The percentages presented in the table are separated by a comma yet in the narrative the percentages are presented with decimal points. For example, career development/continuing education in the table is presented as “135 (37,71)” while the value in the narrative is presented as “37.71”.

The authors further state that “This was followed by factors such as access to technical facilities or infrastructure” yet the table reflects family/personal factors as the second to career development/continuing education.

The narrations presented for table 3 are repeated in both the narration and the discussion section and it was poorly structured and lacked clear organization. The authors should note that the results section should be concise and to the point, highlighting major findings. Additionally, there is no reference made to table 3 in the narration. This places a burden on the reader to locate the corresponding table to which the narration refers.

**Tables 3 and 4**

Tables 3 and 4 both present multivariate analyses; however, there should only be a single multivariate model.

Furthermore, variables that were not significant at bivariate were considered at multivariate analysis for example gender, personal or family reasons and financial reasons.

For both tables 3 and 4, decimal points are presented as commas which is wrong.

The odds ratios for accountability are too high, did the authors test for outliers at analysis stage?

In the paragraph following the figure, the authors refer to “Figure 3” in the statement, “According to the final model (Figure 3)…” However, no figure labeled “Figure 3” exists in the document.

**Figure 1:** Labels for the y-axis are presented in a language that is not English. Furthermore, the figure legend has a variable named “experience rurale” instead of “rural experience”.

From figure 1, rural experience crosses the null which means the variable was not significant, yet the narration implies that rural experience was a significant variable. The authors can justify this by presenting the p-value for this in the final model.

The authors use the term “univariate” to explain the relationship between two variables. This should be renamed as “bivariate”.

**Recommendations and proposed solutions**

These should be presented after the discussion section.

For all recommendations, the authors present the respondents recommendations rather than recommendations as a result of the study. Does this mean that the study had a qualitative aspect to it? If so, this was not mentioned anywhere.

**Discussion**

The first paragraph is not relevant to this section and should be removed.

Overall, the discussion section should be improved without necessarily repeating the results presented in the tables. The discussion should put emphasis on justifying the results obtained in the study.

**Family residence;** The authors should only comment about OR for the multivariate model.

For the statement “This factor was also associated with the choice of practice location in univariate (OR = 3.80) and multivariate analyses (OR = 5.09). This finding aligns with other studies conducted in Botswana [17], Ethiopia [18] and Nepal [19],” it seems incomplete. Does this mean other studies also found a strong positive relationship?

The statement “However, other research, such as that by Liu et al. [20], de Playford et al. [21] et de Grobler et al. [22] found family residence to be less influential..” is also not clear what the authors intended to mean by “less influential.” Additionally, the statement ends with two full stops. This should be edited to one.

**Rural experience;** the authors indicate that there was a relationship between rural experience and the choice of practice location at bivariate analysis; however, this relationship was not significant at multivariate. Apart from this finding being consistent with other studies, what could be the explanation for this?

**Status;** The authors should give details on why they think students are more likely to work in rural areas.

**Financial reasons and personal/family reasons;** the association was positive at bivariate but changed to negative at multivariate analysis. Additionally, aside from comparing the results to other studies, the authors do not explain their findings.

**Accountability and rural origin;** what could be the reason for insignificance at multivariate analysis?

**Gender;** How do these findings relate to other studies?

**Minor revisions**

**Methods**

**Study population:** The authors should use “unregistered physicians” in place of “not registered physicians.”

The authors should move the recommendations to the section after the discussion.

**References:** The authors should translate the references to English.
