# Supplementary material for: Determinants of practice location choices among physicians and medical students in Mali: Insights into addressing medical deserts through evidence-based strategies
Source: PLOS Glob Public Health. 2025 Jun 25;5(6):e0004399. doi: 10.1371/journal.pgph.0004399 (PMC12193920; doi:10.1371/journal.pgph.0004399)
Supplement: Attachment — Submitted filename: REVIEW_PLOSGH_PGPH-D-25-00341.docx [file pgph.0004399.s002.docx]

**REVIEW: Determinants of practice location choices among physicians and medical students in Mali: insights into addressing medical deserts through evidence-based strategies**--Issa Kalossi, MD, MPH, et al

General:

This paper describes conducted a cross-sectional and prospective study targeting doctors and final-year medical students in Mali. The purpose of the study was to identify factors influencing doctors' workplace decisions and identify factors that may be relevant to addressing workforce shortages of medical doctors in this setting.

The reviewer appreciated the design of the survey, however the random section of 358 respondents was confusing. Why was the entire population of respondents not considered and why were specifically 358 responses randomly selected?

This reviewer is curious about why medical doctors were identified as the target population when many diverse professionals (nurses, laboratorians, therapists, etc.) make healthcare delivery possible. The narrowing of this definition of medical professionals could be described in the introduction and discussion.

This reviewer suggests sharing the survey in supplemental material.

Specific comments:

Results. It is customary in survey responses to provide the response rate (responses/total population surveyed). Kindly provide the response rate.

Tables: The asterisks signifying statistical significance were not reflected in the table, despite being defined in the key. This reviewer suggests updating the tables with these symbols.

Figure 1: The regression model contained French (oui/non) responses. For clarity, this reviewer suggests a consistent language throughout.

Discussion: The authors observe a gender imbalance in their response demographics. This should also be described as a limitation of findings between male and female respondents. For example, did the female candidates opt out of responding to the survey or does this represent the underlying distribution of male to female medical doctors? Could purposive sampling of the respondents to enrich female participants to better describe differences?
