# Supplementary material for: Determinants of practice location choices among physicians and medical students in Mali: Insights into addressing medical deserts through evidence-based strategies
Source: PLOS Glob Public Health. 2025 Jun 25;5(6):e0004399. doi: 10.1371/journal.pgph.0004399 (PMC12193920; doi:10.1371/journal.pgph.0004399)
Supplement: Attachment — Submitted filename: Response to Reviewers.docx [file pgph.0004399.s003.docx]

Response to Reviewers

Journal requirement

1. We do not publish any copyright or trademark symbols that usually accompany proprietary names, eg (R), (C), or TM (e.g. next to drug or reagent names). Please remove all instances of trademark/copyright symbols throughout the text, including ® on page 2.

Trademark remove on page 2

2. Please upload a copy of Figures 1 to 3 which you refer to in your text on page 7. Or, if the figure is no longer to be included as part of the submission please remove all reference to it within the text.

Figure 1-2 are no longer to be included, so the order number has been corrected (now figure 1) on page 7

Reviewers’ comments

Reviewer 1 :

Abstract:

Abstract section broke into different part on page 1

95% CI standardized all over the manuscript and suggestions considered

Introduction:

Sentences have restructured on page one and 2

The Hart’s law has been briefly explained on the previous sentence, so we did think it may be redundant

Methods:

We choose final years because they are the closest group of students to the professional world and they might have a clear vision on their future practice location choice.

Different component of the formula defined on page 2

The reliability of the questionnaire was not formally assessed using Cronbach’s alpha. However, the questions were carefully designed to align with established literature and reviewed by subject matter experts to ensure conceptual consistency. For future studies, we will consider performing a formal reliability analysis.

After the pilot test they were few changes, some minor changes had been made to its clarity and its ease of use.

Result:

Ensure consistency in reporting statistical results. For instance, some p-values are explicitly stated (e.g., p < 0.001), while others are embedded within the text. Consider using a uniform format. Odds ratios (ORs) should always include confidence intervals (CIs) for clarity, which is well done in most instances. However, for the final model (Figure 3), it would be helpful to report specific ORs for each significant factor.

The CIs were correct all over the manuscript and Ors were added to the figure 3 now figure 1

The average age and standard deviation are useful, but it would be beneficial to include a median age as well, considering the potential skewness of professional experience. Since gender distribution is highly imbalanced (83.80% male), a brief mention of whether this reflects the broader physician/student demographic in Mali would provide a better context.

This suggestion has been considered on page 12

The fact that rural origin was significant in univariate analysis but not in multivariate analysis suggests confounding variables. Consider briefly explaining why this might be the case. The term "accountability" as a motivation should be clearly defined. What specific aspects of accountability motivate respondents? Ethical obligations? Community responsibility?

Suggestion considered on page 5

We considered accountability toward the family and community, so it’s a community accountability in this manuscript.

Discussion:

The contradiction between Liu et al. (2020) and other studies regarding the role of family residence could be explained further. Does it reflect cultural differences, economic factors, or healthcare policies?

Explained on page 9

The study does not explicitly mention potential biases (e.g., self-selection bias, social desirability bias in responses). These should be acknowledged. Since 51.22% of respondents work in Bamako, the findings may not fully represent rural physicians. Consider discussing this limitation.

Social desirability biases have been acknowledged. However, we did not acknowledge self-selection biases because respondents have been randomly selected.

Reviewer 2

1. Who bears the cost of medical education in the country and what is the contribution of students and doctors towards that cost? [the reason for this info is that very often when people pay for their education, they would want to recoup the costs from their pocket as soon as possible]

In Mali, medical studies are free of charge, the government ensure all the cost and whithout any payback studies. In addition, students even have a monthly stipend around 40€.

2. What proportion of doctors are in the private sector and what proportion in the public sector? Is the sample proportionate to these realities? Who are the major employers other than the government? What about those in the NGOs?

Public sector employs only 58 doctors among our respondents versus 188 for private sectors. Private sector includes NGOs and private clinics

3. Any information or previous study that gives an understanding of who is preferred for medical consultations on the part of the community?

4. Finally, any information on HRH analysis in terms of projections for the country by urban and rural populations.

At this point, there is no official HRH analysis that comes to our attention even though we think it very important for a country like Mali to have such a plan.

Reviewer 3:

This paper describes the determinants of practice location choices among physicians and medical students in Mali. It should be publishable provided some extra work is done on the results and discussion section. The methods section and presentation of the results were not satisfactorily done and the interpretation of the results did not provide adequate depth. Additionally, there was no consistency in terminology with different terms being used interchangeably to refer to the same concept. There is a disparity where the authors use the term physician in the title and the term doctors throughout the study. The authors should rewrite their article to use either of the mentioned terms ie either physicians or doctors.

This point has been considered all aver the manuscript

In paragraph 2, the authors state that “Nationally, only 32% of these centers have a physician, compared to 98% in Bamako, while the figure drops to just 29% in the rest of the country” Does this mean that only 32% of community health centers have a physician. And of these physicians 98% work in Bamako? What does the 29% in the rest of the country mean? These statistics are not clear and make it harder for the reader to understand the message.

32% in the global percentage nation wide

And if we disaggregate, 98% is for the community health centers in Bamako and 29% for other regions.

Sampling: There were two distinct categories of participants, was simple random sampling the most appropriate sampling method for this study?

In total, we had 3011 registered physicians and 415 final year students (3426 people). All the study population has been combined to make one single list. Then, we performed a simple random sampling so that every subject has the same chance to be selected.

In the methods sections the dependent variable is named “choice of practice location”, the table uses “practice location” while the narrative is “preferred practice location”. Do these mean the same thing?

“Practice location” is the actual practice location

“Preferred practice location” is the place the respondents would choose if they are given the choice.

The authors mention the following variables however these are not presented in the table namely; age and professional experience. The authors further mention that “among the 358 respondents, 191 (53.35%) were born and/or raised in urban areas” yet the table has a variable named “rural origins”, do these mean the same thing?

We confirm that these appellations are the same, respondents who declared to be born or raised in rural area were considered to have rural experience.

The authors state that “When asked about their preferred practice location, less than 38% (135/358) of respondents indicated a preference for working in rural areas if given the choice”. Yet the results in the table for practice location reflect 22% as the value for preference for working in rural areas. Why did the authors choose to use 38%?

We confirm that 135 over 358 indicate the rural area as preferred practice location (37.71% more precisely) cf. table 3

Reviewer 4:

1. Expand the introduction to better connect Mali's healthcare challenges to the broader global rural healthcare crisis. Adding insights on Mali’s healthcare infrastructure, workforce distribution, and medical education system would provide necessary context.

Introduction section expanded and medical studies aspects has been added on page 2

2. The introduction should state the gap in literature more concisely and directly link this to the study objectives.

Introduction section rewrote and this suggestion has been considered

3. The references are heavily reliant on older studies. Incorporating more recent (post-2020) references would improve the manuscript’s relevance.

This is because our subject is not very widely treated mainly in our context. This is why we before 2020 references in manuscript.

4. For methods, specifically authors must justify the chosen 2% refusal rate assumption in the sample size calculation.

This rate has been chosen prior the study because similar studies did not specifically treat this aspect. And the post study rate was 5.8%. page 4

5. Please provide a clear rationale for excluding non-thesis-year students and non-registered physicians.

We choose final years because they are the closest group of students to the professional word and they might have a clear vision on their future practice location choice.

About the non-registered physicians, it’s because the sampling was performed on the national medical council’s list.

6. Authros should clarify whether the questionnaire was pilot-tested. If so, describe what modifications were made based on pilot results.

Comment solved on page 3

7. Highly recommend incorporating visual aids such as bar charts, pie graphs, or infographics to present key findings more effectively.

Considering the number and types of variables we had; we found it very difficult using pie charts or bars. Reason why results have been summarized into tables

8. While Odds Ratios (ORs) and Confidence Intervals (CIs) are reported, a brief interpretation of these results for non-statistical readers would improve clarity.

We will add comments ORs in the result section

9. Since male respondents dominate the sample (83.8%), the potential impact of gender bias on findings should be explicitly discussed.

Gender imbalance discussed on page 12

Reviewer 5:

The reviewer appreciated the design of the survey, however the random section of 358 respondents was confusing. Why was the entire population of respondents not considered and why were specifically 358 responses randomly selected?

In total, we had 3011 registered physicians and 415 final year students (3426 people). We sampled 358 respondents out of this list. Surveying the entire population would very heavy in terms of budget and time, that why choose a scientifically accepted method of sampling.

This reviewer is curious about why medical doctors were identified as the target population when many diverse professionals (nurses, laboratorians, therapists, etc.) make healthcare delivery possible. The narrowing of this definition of medical professionals could be described in the introduction and discussion.

We acknowledge the importance of studying all the health system actors but knowing our context this study will be very welcomed and may be a prelude of future studies regarding this matter.

Discussion: The authors observe a gender imbalance in their response demographics. This should also be described as a limitation of findings between male and female respondents. For example, did the female candidates opt out of responding to the survey or does this represent the underlying distribution of male to female medical doctors? Could purposive sampling of the respondents to enrich female participants to better describe differences?

The gender imbalance has been discussed on page 12

Gender; How do these findings relate to other studies?

In the literature we reviewed, gender imbalance was not specifically reported
