# Supplementary material for: Determinants of practice location choices among physicians and medical students in Mali: Insights into addressing medical deserts through evidence-based strategies
Source: PLOS Glob Public Health. 2025 Jun 25;5(6):e0004399. doi: 10.1371/journal.pgph.0004399 (PMC12193920; doi:10.1371/journal.pgph.0004399)
Supplement: Attachment — Submitted filename: Determinants of practice location choices-Re-review.docx [file pgph.0004399.s004.docx]

**Reviewer’s report**

**Study title:** Determinants of practice location choices among physicians and medical students in Mali: insights into addressing medical deserts through evidence-based strategies

**Version:** 1.0	**Date:** 8^th^ May 2025

**Reviewer:** Sheillah Ansiima

**Abstract:** The methods and conclusion section of the abstract are not satisfactorily address. Takeaways have not been emphasized.

**Introduction: This section has not been sufficiently addressed.**

The sentence **“**Which is a global concern that poses significant challenges to equitable healthcare delivery” seems to be a continuation of the first statement and should be rephrased as such.

The initial comment “In paragraph 2, the authors state that “Nationally, only 32% of these centers have a physician, compared to 98% in Bamako, while the figure drops to just 29% in the rest of the country” Does this mean that only 32% of community health centers have a physician. And of these physicians 98% work in Bamako? What does the 29% in the rest of the country mean? These statistics are not clear and make it harder for the reader to understand the message.” still stands.

**Methods;** The comment was not addressed.

**Data collection:** The comment **“The authors do not mention the type of questionnaires used. Furthermore, the authors do not mention whether the pre-tested students and physicians included in the pre-test were included in the final study”** was not addressed.

Statistical analysis was appropriately addressed

**Results**

The comment “In the methods sections the dependent variable is named “choice of practice location”, the table uses “practice location” while the narrative is “preferred practice location”. Do these mean the same thing?” was not addressed. Kindly align to have consistent name.

Table 1: Decimal points are still presented as commas

**Results Paragraph 1:** The comments below were not addressed.

1. The authors mention the following variables however these are not presented in the table namely; age and professional experience.” Kindly include these variables in the table.

Table 1 reflects 68% as the percentage representing physicians yet the authors state that “physicians constituted over 68%”. Rephrase to say “physicians constituted 68.72% of the”

“77% of the physicians working in the private sector” is not reflected in table 1.This statement includes only the physician yet the results mean that of all respondents, 77% work in the private sector. Rephrase to say “77% of the respondents are working in the private sector”

“More than 69% of respondents reported no prior experience working or interning in rural settings” why didn’t the authors use the actual percentage presented in the table? Kindly rephrase to “About 69% of the respondents reported…”

**Paragraph 3:** The comment not addressed

“The authors further state that “This was followed by factors such as access to technical facilities or infrastructure” yet the table reflects family/personal factors as the second to career development/continuing education” was not addressed. Rephrase the statement to “This was followed by family/personal factors”

Table 1,3 and 4 are still presented with commas in place of decimal points.

The comment “The narrations presented for table 3 are repeated in both the narration and the discussion section and it was poorly structured and lacked clear organization. The authors should note that the results section should be concise and to the point, highlighting major findings. Additionally, there is no reference made to table 3 in the narration. This places a burden on the reader to locate the corresponding table to which the narration refers” was not addressed.

**Tables 3 and 4;** the following comments were not addressed.

Tables 3 and 4 both present multivariate analyses; however, there should only be a single multivariate model.

Furthermore, variables that were not significant at bivariate were considered at multivariate analysis for example gender, personal or family reasons and financial reasons. Factors that are not significant at univariate (i.e. p-value >0.2) should not be included in the multivariate model.

Figure 1: The comment “Furthermore, the figure legend has a variable named “experience rurale” instead of “rural experience” as not been addressed.

The authors use the term “univariate” to explain the relationship between two variables. This should be renamed as “bivariate”. This has not been addressed.

**Recommendations and proposed solutions: The following comments have not been addressed.**

These should be presented after the discussion section.

For all recommendations, the authors present the respondents recommendations rather than recommendations as a result of the study. Does this mean that the study had a qualitative aspect to it? If so, this was not mentioned anywhere.

Comments related to the discussion are not addressed

**Rural experience;** the authors indicate that there was a relationship between rural experience and the choice of practice location at bivariate analysis; however, this relationship was not significant at multivariate. Apart from this finding being consistent with other studies, what could be the explanation for this?

**Status;** The authors should give details on why they think students are more likely to work in rural areas.

**Financial reasons and personal/family reasons;** the association was positive at bivariate but changed to negative at multivariate analysis. Additionally, aside from comparing the results to other studies, the authors do not explain their findings.

**Accountability and rural origin;** what could be the reason for insignificance at multivariate analysis?

**Gender;** How do these findings relate to other studies?
