# Supplementary material for: Determinants of practice location choices among physicians and medical students in Mali: Insights into addressing medical deserts through evidence-based strategies
Source: PLOS Glob Public Health. 2025 Jun 25;5(6):e0004399. doi: 10.1371/journal.pgph.0004399 (PMC12193920; doi:10.1371/journal.pgph.0004399)
Supplement: Attachment — Submitted filename: Response_to_Reviewers_auresp_2.docx [file pgph.0004399.s005.docx]

**Response to Reviewers**

**We would like to sincerely apologize for having overlooked these important comments in our previous response.**

**Abstract:** The methods and conclusion section of the abstract are not satisfactorily address. Takeaways have not been emphasized.

These sections have been adjusted.

**Introduction: This section has not been sufficiently addressed.**

The sentence **“**Which is a global concern that poses significant challenges to equitable healthcare delivery” seems to be a continuation of the first statement and should be rephrased as such.

This comment has been addressed.

The initial comment “In paragraph 2, the authors state that “Nationally, only 32% of these centers have a physician, compared to 98% in Bamako, while the figure drops to just 29% in the rest of the country” Does this mean that only 32% of community health centers have a physician. And of these physicians 98% work in Bamako? What does the 29% in the rest of the country mean? These statistics are not clear and make it harder for the reader to understand the message.” still stands.

To clarify:

- The figure "32%" refers to the national average: only 32% of community health centers (CSCOMs) across Mali have a physician.
- Among these, 98% of CSCOMs in Bamako have a physician, indicating a strong concentration of medical doctors in the capital.
- In contrast, only 29% of CSCOMs outside Bamako are staffed with a physician, highlighting a significant disparity between the capital and the rest of the country.

The sentence has been rephrased in the manuscript as well.

**Methods:** The comment was not addressed.

**Data collection:** The comment **“The authors do not mention the type of questionnaires used. Furthermore, the authors do not mention whether the pre-tested students and physicians included in the pre-test were included in the final study”** was not addressed.

These participants were not included in the final sample, and it has been mentioned in the manuscript.

Statistical analysis was appropriately addressed

Thanks!

**Results**

The comment “In the methods sections the dependent variable is named “choice of practice location”, the table uses “practice location” while the narrative is “preferred practice location”. Do these mean the same thing?” was not addressed. Kindly align to have consistent name.

“Practice location” is the actual practice location (they either work in Bamako or in the other regions)

“Choice of practice location” and “Preferred practice location” mean the same thing : this means the place the respondents would choose if they were given the choice.

It has been addressed in the manuscript.

Table 1: Decimal points are still presented as commas

The comment has been addressed in the manuscript.

**Results Paragraph 1:** The comments below were not addressed.

1. The authors mention the following variables however these are not presented in the table namely; age and professional experience.” Kindly include these variables in the table.

These lines have been added in the table1

Table 1 reflects 68% as the percentage representing physicians yet the authors state that “physicians constituted over 68%”. Rephrase to say “physicians constituted 68.72% of the”

The sentence has been rephrased.

“77% of the physicians working in the private sector” is not reflected in table 1.This statement includes only the physician yet the results mean that of all respondents, 77% work in the private sector. Rephrase to say “77% of the respondents are working in the private sector”

We would like to clarify that the statement "77% of the physicians working in the private sector" refers specifically to the 246 physicians included in the study, not to all respondents.

For more clarity, we rephrased the sentence in the manuscript.

“More than 69% of respondents reported no prior experience working or interning in rural settings” why didn’t the authors use the actual percentage presented in the table? Kindly rephrase to “About 69% of the respondents reported…”

The comment has been considered

**Paragraph 3:** The comment not addressed

“The authors further state that “This was followed by factors such as access to technical facilities or infrastructure” yet the table reflects family/personal factors as the second to career development/continuing education” was not addressed. Rephrase the statement to “This was followed by family/personal factors”

In fact, family/personal factors were the second most cited reason after career development/continuing education.

It’s been corrected in the manuscript.

Table 1,3 and 4 are still presented with commas in place of decimal points.

Addressed.

The comment “The narrations presented for table 3 are repeated in both the narration and the discussion section and it was poorly structured and lacked clear organization. The authors should note that the results section should be concise and to the point, highlighting major findings. Additionally, there is no reference made to table 3 in the narration. This places a burden on the reader to locate the corresponding table to which the narration refers” was not addressed.

All the section about table 4 has been restructured in the manuscript

**Tables 3 and 4;** the following comments were not addressed.

Tables 3 and 4 both present multivariate analyses; however, there should only be a single multivariate model.

We would like to clarify that although both Table 3 and Table 4 present results from multivariate analyses, they are based on distinct models exploring different levels of determinants (sociodemographic and professional factors)

Regarding the inclusion of univariate analyses, we believe it is important to present them alongside multivariate results to allow readers to:

- Understand the crude associations before adjustment,
- See how relationships change after controlling for potential confounders,
- Identify variables that were initially significant but lost significance in the adjusted model, providing insights into the structure of the relationships.

Furthermore, variables that were not significant at bivariate were considered at multivariate analysis for example gender, personal or family reasons and financial reasons. Factors that are not significant at univariate (i.e. p-value >0.2) should not be included in the multivariate model.

We included variables such as sex and personal/family reason in the multivariate models not solely based on their significance in univariate analysis, but because they are widely reported in the literature as important determinants of physicians’ decision-making regarding practice location.

Their inclusion in the model allows us to:

- Control for potential confounding effects of these key variables,
- And ensure comparability with findings from similar studies

Figure 1: The comment “Furthermore, the figure legend has a variable named “experience rurale” instead of “rural experience” as not been addressed.

This comment has been addressed

The authors use the term “univariate” to explain the relationship between two variables. This should be renamed as “bivariate”. This has not been addressed.

We acknowledge the distinction between univariate and bivariate analysis from a statistical standpoint. However, in the context of epidemiological and public health research, it is a common practice to refer to analyses that assess the relationship between a single independent variable and the outcome, typically using logistic regression as "univariate analysis", even though this technically involves two variables !

**Recommendations and proposed solutions: The following comments have not been addressed.**

These should be presented after the discussion section.

For all recommendations, the authors present the respondents recommendations rather than recommendations as a result of the study. Does this mean that the study had a qualitative aspect to it? If so, this was not mentioned anywhere.

We would like to clarify that was not a qualitative component of the study. Rather, it was based on a single open-ended question included at the end of the structured questionnaire:

“What would you recommend to national authorities to ensure a fair distribution of physicians across the country?”

This question was intended to complement the quantitative findings by capturing respondents’ perspectives in their own words and was not treated as a separate qualitative arm of the study.

Comments related to the discussion are not addressed

**Rural experience;** the authors indicate that there was a relationship between rural experience and the choice of practice location at bivariate analysis; however, this relationship was not significant at multivariate. Apart from this finding being consistent with other studies, what could be the explanation for this?

Rural experience may correlate with other variables like rural origin, family location, or personal motivations, which could dilute its independent effect in the adjusted model.

**Status;** The authors should give details on why they think students are more likely to work in rural areas.

Medical students often display a higher level of idealism and willingness to serve underserved populations, as documented in other studies. At this stage, they may be more receptive to rural practice, especially if they perceive it as a meaningful opportunity for impact.

**Financial reasons and personal/family reasons;** the association was positive at bivariate but changed to negative at multivariate analysis. Additionally, aside from comparing the results to other studies, the authors do not explain their findings.

The paragraph has been adjusted in the manuscript

**Accountability and rural origin;** what could be the reason for insignificance at multivariate analysis?

This has been considered and the chapiter “Accountability” has been updated accordingly.

**Gender;** How do these findings relate to other studies?

We acknowledge the importance of considering other studies, and the manuscript has updated accordingly.

**Journal Requirements:**

For reference check

Reference 3 : the reference stand, but the link has been updated, and it’s been changed in the manuscript
